# Complicated Appendicitis Associated With Appendiceal Mucinous Neoplasm Within Amyand’s Hernia in a Patient With Previous Hernia Repair: A Case Report and Literature Review

**DOI:** 10.7759/cureus.65801

**Published:** 2024-07-30

**Authors:** Carlos A Navarro-Castañeda, Luis A Pérez-Silva, Rubén A Sandoval-Barba, Nubia A Ramírez-Buensuceso-Conde, Jorge Farell-Rivas

**Affiliations:** 1 General Surgery, Hospital Central Sur de Alta Especialidad, Mexico City, MEX; 2 General Surgery, Hospital Español, Mexico City, MEX

**Keywords:** case report, perforated appendicitis, appendiceal neoplasms, amyand’s hernia, inguinal hernia

## Abstract

Amyand's hernia is defined as the presence of the appendix within an inguinal hernia sac, which is often associated with appendicitis. The association of an Amyand's hernia with an appendicular tumor has been reported in very few cases. This case report presents a 67-year-old female patient who came to the emergency department with symptoms indicative of a complicated inguinal hernia. Following surgical treatment, the diagnosis of Amyand's hernia with cecal perforation associated with an appendicular tumor was established in the context of a previous laparoscopic femoral hernia repair. The combination of these conditions has not been previously reported. The presentation of this case provides data on the clinical presentation, diagnosis, and treatment of this rare pathology that requires a high clinical suspicion to achieve a preoperative diagnosis.

## Introduction

The presence of an appendix with appendicitis inside of an inguinal hernia sac is known as “Amyand’s hernia” (AH), named after Claudius Amyand, the first surgeon who described and treated it [1]. It is a rare entity, with large retrospective studies reporting an incidence of 0.07-0.13%, with a bimodal age distribution first in infants (one month to one year) and second in elderly patients (70 years) [2]. Neoplasm of the appendix is another rare occurrence, with an incidence between 0.58% and 1.68% of appendectomy specimens [3,4]. The presence of both of these pathologies is even more infrequent, with very few cases reported in the literature. We report the case of a 67-year-old female who presented with an incarcerated hernia, which was AH, that later was reported with a mucinous neoplasm of the appendix.

## Case presentation

**Figure 1:**
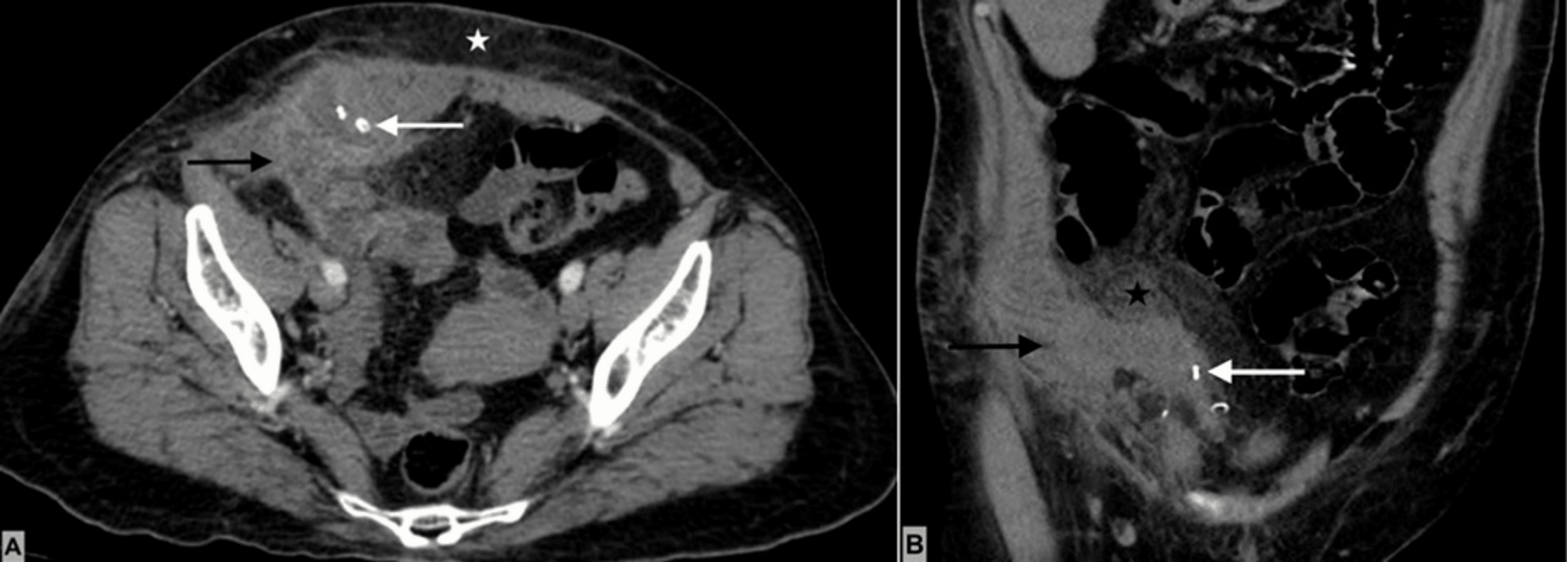
Contrast-enhanced abdominal tomography A: Axial section showing a right inguinal hernia containing appendix (Amyand’s hernia) with diffuse wall thickening and enhancement (solid black arrow) and periappendicular fluid. There is also evidence of stranding of subcutaneous fat tissue (white star), and the tackers placed on the previous surgery are visible (solid white arrow). B: Coronal section showing a right Amyand’s hernia with diffuse periappendicular inflammatory changes (black solid arrow), tackers placed on previous surgery (solid white arrow), and diffuse intraabdominal fat stranding (solid black star).

A 67-year-old patient came to the emergency department with abdominal pain that has persisted for the last 20 days. Past medical history without any chronic disease. The patient has a history of cesarean section, laparoscopic cholecystectomy, and laparoscopic inguinal hernia repair for femoral hernia. Vital signs were normal. Abdominal pain was localized to the right lower quadrant, associated with a palpable painful mass of 10 × 8 cm in the same area. No signs of peritoneal irritation were present. Laboratory results revealed leukocytosis (11.5 × 10^9^/L) and neutrophils at 67.8%; the rest of the parameters were within the normal range. Contrast-enhanced computed tomography revealed a hernia defect in the lower right quadrant of the abdominal wall with a non-reducible small bowel, as well as stranding of mesenteric fat and overlying subcutaneous tissue. Prosthetic material (mesh and metallic tackers) was observed in the right inguinal region (Figure 1).

**Figure 2:**
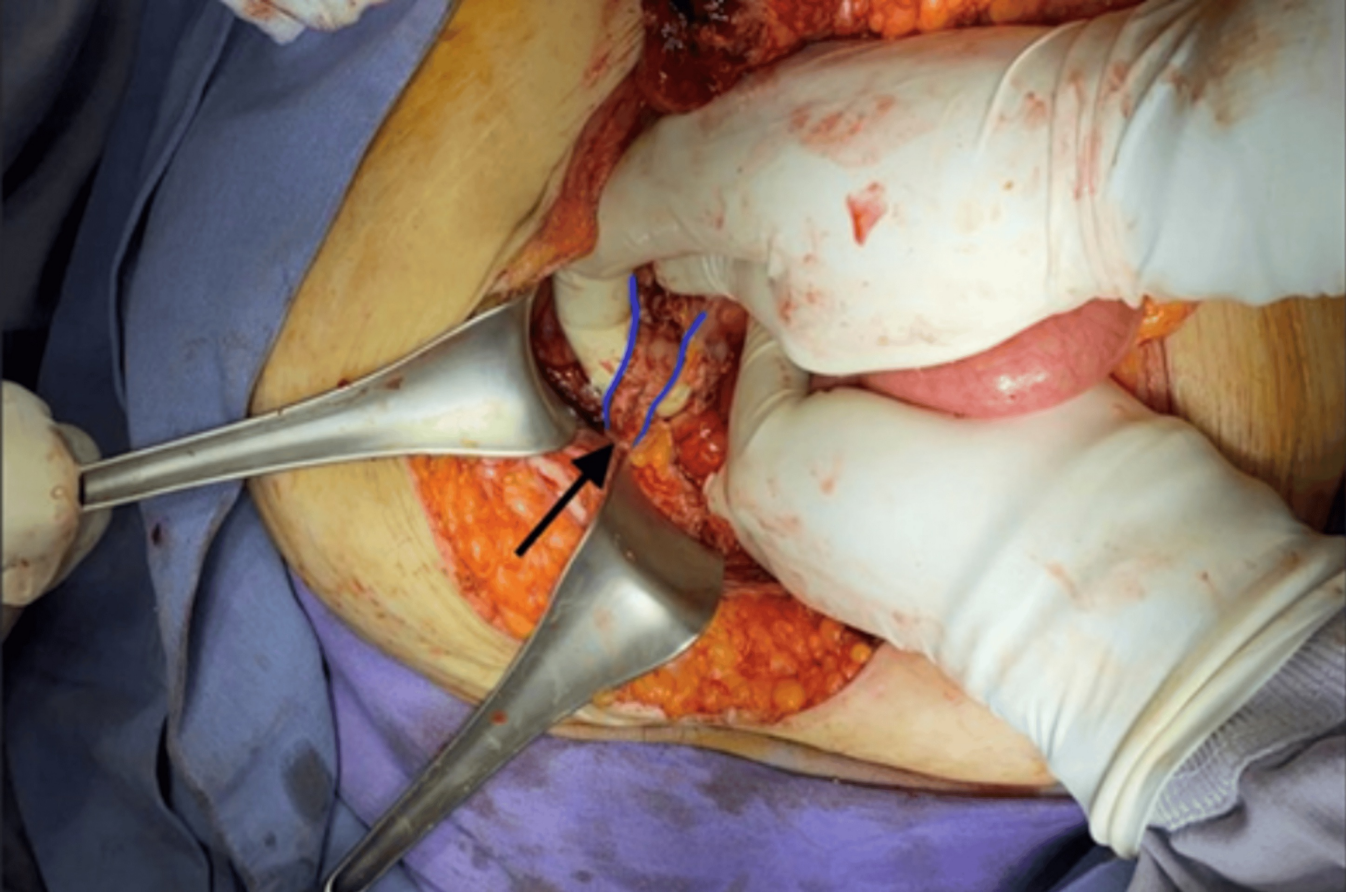
Intraoperative image of Amyand’s hernia Indirect hernia containing the tip of a perforated appendix (black solid arrow). The appendix is outlined in blue, and the perforation at the base of the appendix can be observed beneath the surgeon’s right thumb.

**Figure 3:**
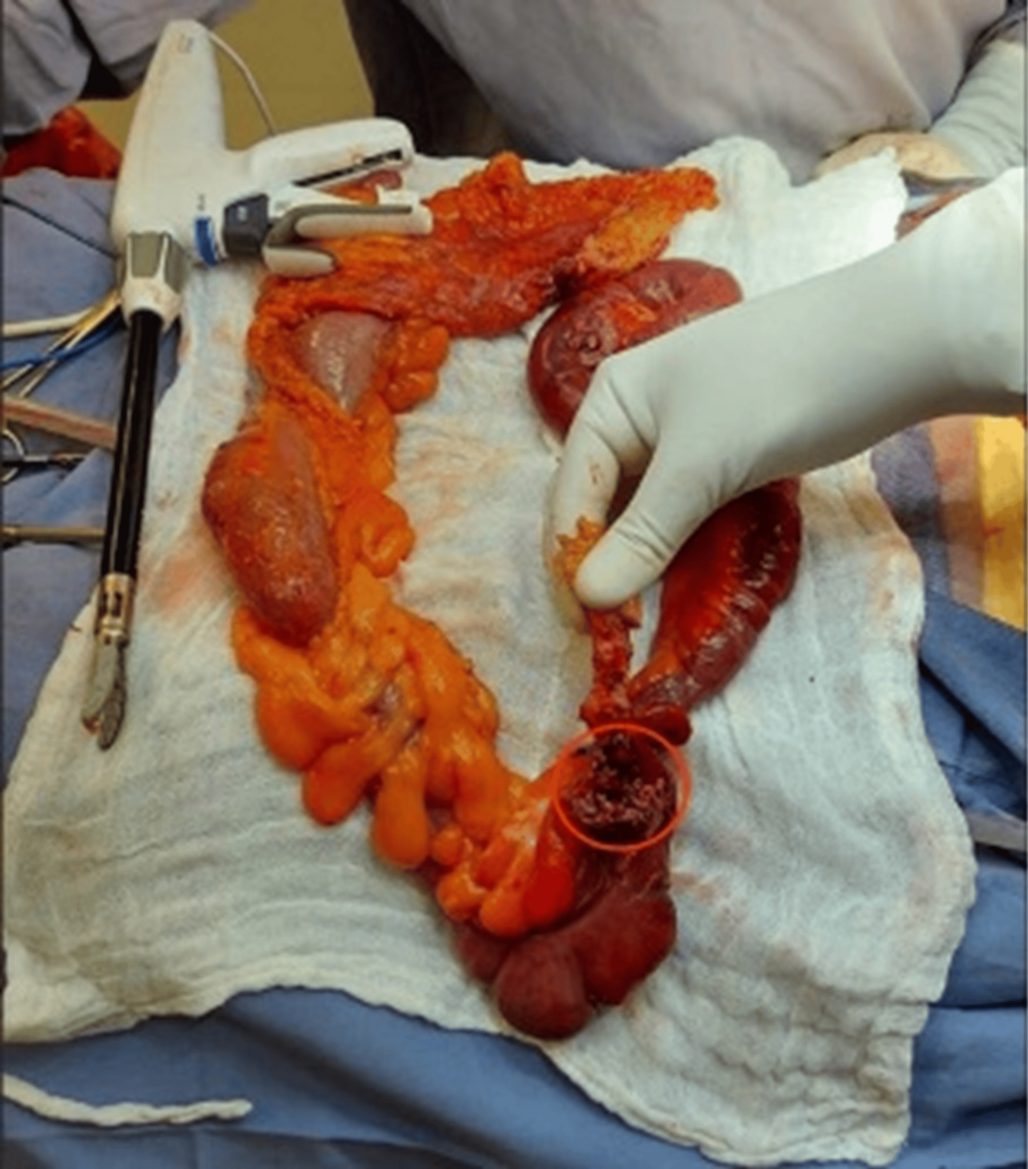
Product of right hemicolectomy with perforated appendix It shows the appendix in the hand of the surgeon; there is evidence of an ulcerative exophytic lesion that caused perforation at the base of the appendix (red circle).

Laparotomy was performed with the following findings: right indirect inguinal hernia contains the appendix (Figure 2), showing signs of acute appendicitis with perforation of the appendiceal base and involvement of the cecal wall (Figure 3). A 20-mL peri-appendiceal abscess within the hernia sac was drained. The terminal ileum was adhered to the cecum, with ischemic changes in the last 30 cm. An extended right hemicolectomy was performed and ileo-transverse mechanical side-to-side anastomosis was carried out. The hernia defect was closed with a continuous suture using polypropylene. Broad-spectrum antibiotics and analgesics were continued.

Oral liquids were initiated within the first 24 hours, progressing to a soft diet on the second postoperative day. Enterococcus faecalis was reported from the culture of the abscess in the subcutaneous tissue of the right lower quadrant, for which targeted antibiotic treatment was administered during hospitalization. The patient was discharged on the seventh postoperative day. During outpatient follow-up, a seroma without signs of infection was diagnosed at the wound site, resolving with local care. Abdominal drainage was removed on the 10th postoperative day, with the last output of 20 mL in 24 hours of serous aspect.

**Figure 4:**
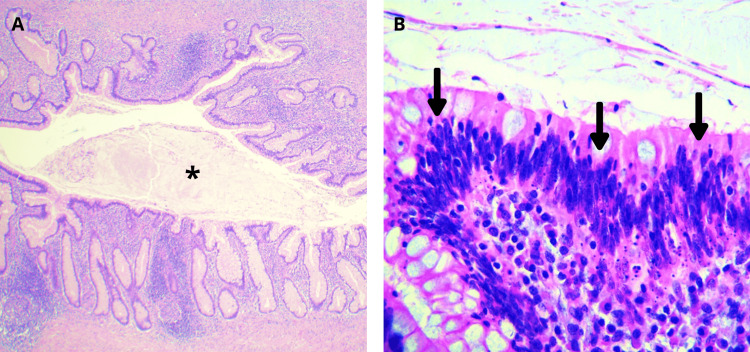
Microscopic image of the appendix A: Intraluminal mucin (black asterisk) within a dilated appendix (hematoxylin and eosin; 40×). B: Appendiceal mucosa displaying areas of low-grade dysplasia (black arrows) (hematoxylin and eosin; 320×).

The pathology report revealed a low-grade appendiceal mucinous neoplasm (LAMN) (Figure 4), perforation with intense fibrosis, and acute and chronic inflammation consistent with a foreign body reaction. Angiodysplasias were noted. Ten lymph nodes demonstrated mixed hyperplasia.

The patient was evaluated by medical oncology, establishing clinical stage 0 (Tis LAMN N0 M0), therefore continuing surveillance without the need for oncologic treatment. A follow-up colonoscopy at three months showed no neoplasms or anastomotic complications. A biopsy of the anastomotic site revealed no malignancy. During the most recent medical consultation, four months after surgery, the patient exhibited no complications and displayed no clinical signs of tumor activity.

## Discussion

AH is a rare condition, with an incidence ranging from 0.14 to 1.3% of all inguinal hernias, and its association with acute appendicitis occurs only in 0.07 to 0.13% of inguinal hernias [2]. This hernia type predominantly affects males, constituting 83-90% of cases [2,5]. A bimodal age distribution has been observed, with occurrences in individuals under one year and those over 70 years and an average age of diagnosis of 58.5 ± 19.6 years [5].

While the diagnosis of AH can be made during elective surgeries for uncomplicated inguinal hernias or incidentally in imaging studies, most cases are diagnosed in emergency situations (63.57%). Preoperatively, 76.2% of cases undergoing emergency surgery are diagnosed as complicated hernias (incarcerated or strangulated) [2]. According to a systematic review on surgical decision-making in patients with AH [2], 94.2% of elective surgeries and 90.4% of emergency surgeries were performed through an open approach. In adults undergoing elective surgery, appendectomy was performed in 65% of cases, in contrast to 90% in emergency surgeries. Mesh utilization in elective inguinal hernia repairs associated with appendectomy was 62%, while in emergency surgeries mesh was employed in only 19% of cases.

Appendiceal tumors are also rare, occurring in 0.9-2% of appendectomy specimens [3,4,6]. Multiple histological patterns have been described for appendiceal neoplasms. Current classification encompasses mucinous neoplasms, neuroendocrine tumors, goblet cell adenocarcinoma, non-mucinous adenocarcinoma, and signet ring cell adenocarcinoma [7]. Depending on the study methodologies, mucinous neoplasms and neuroendocrine tumors are the most common appendiceal neoplasms. Mucinous neoplasms include LAMN, high-grade mucinous neoplasms, and mucinous adenocarcinoma. LAMNs are well-differentiated adenomas with the potential for malignant behavior and spread beyond the appendix [6]. This histology includes the previously termed mucinous cystic neoplasms and mucinous cystadenomas.

Patients with LAMN limited to the appendix who undergo either appendectomy or right hemicolectomy have a 95% five-year disease-free survival, making appendectomy alone appropriate for management [6]. However, in cases where the neoplasm corresponds to a mucinous adenocarcinoma limited to the appendix, right hemicolectomy is indicated. For patients with any appendiceal mucinous neoplasm associated with peritoneal metastasis or pseudomyxoma peritonei, the therapeutic strategy leans toward more extensive surgeries to achieve cytoreduction. These interventions may or may not include intraperitoneal chemotherapy [6].

The coexistence of two rare conditions, AH and appendiceal tumors, represents an exceedingly rare clinical scenario. Some review articles have identified between seven and nine published clinical cases with the diagnosis of AH alongside the presence of appendiceal neoplasms [5,8]. We conducted a search on Google Scholar and PubMed databases for reported cases of AH with appendiceal tumors published from January 2018 to November 2023. We found 11 patients [9-19], with only one of these cases having been cited in previous reviews. The main characteristics of the reported cases are summarized in Table 1.

**Table 1:** Diagnostic and therapeutic characteristics of reported cases of Amyand’s hernia with appendiceal neoplasms from 2018 to 2023 LAMN: low-grade appendiceal mucinous neoplasm; NET: neuroendocrine tumor

Article, year	Age (years)/sex	Emergency or elective surgery	Surgical approach	Type hernia repair	Surgical approach appendix	Appendicitis classification	Histopatology	Colon neoplasia associated
Zarbaliyeb et. al. 2018 [9]	54/female	Emergency	Open	Primary closure	Appendicectomy	-	LAMN	N/A
Oh et. al. 2018 [10]	37/male	Emergency	Laparoscopic	Primary closure	Appendicectomy	Non-complicated	LAMN	N/A
Sarici et. al. 2019 [11]	64/male	Emergency	Open	Tension repair	Appendicectomy + right hemicolectomy (second surgery)	Non-complicated	Low-grade NET	No
Russler et. al. 2019 [12]	70/male	Emergency	Laparoscopic/open	Tension repair	Appendicectomy	Complicated	Diffuse large B-cell lymphoma	No
Allué et. al. 2019 [13]	84/male	Elective	Open	Lichtenstein	Appendicectomy	-	LAMN	No
Grez et. al. 2020 [14]	71/male	Emergency	Open	Lichtenstein (second surgery)	Appendicectomy	Complicated	Carcinoid (NET)	No
Oyelowo et. al. 2020 [15]	28/male	Elective	Open	Nylon-Darn	Appendicectomy	-	Fibroma	N/A
Aabo et. al. 2021 [16]	67/male	Elective	Open	Lichtenstein	Appendicectomy	-	Carcinoid (NET)	N/A
Fiordaliso et. al. 2021 [17]	87/male	Emergency	Open	Bassini	Ileocecal resection	Non-complicated	Adenocarcinoma	No
Arenas et. al. 2022 [18]	72/male	Elective	Open	Tension-free repair	Appendicectomy	-	LAMN	No

Most patients were male (81.8%), with ages ranging from 28 to 87 years old (mean age of 64.45 years). The case we present falls within the expected age range but is female, a rarity in previously documented cases. Six were managed as emergent scenarios, whereas five underwent elective surgeries, differing from cases reported in other reviews where the majority underwent emergency surgeries [8]. Laparoscopic approaches were employed in only two cases [10,12]. In five patients, inguinal hernia repair incorporated the use of mesh, while primary closure was performed in the remaining cases. One case necessitated inguinal mesh repair during a subsequent surgical intervention [14]. None of the cases with inguinal mesh repair reported mesh infection.

In all cases, resection of the appendiceal tumor was performed. Appendectomy alone sufficed in 81.8% of instances. In two cases, colonic resection was also performed, one involving a neuroendocrine tumor [11] and the other with adenocarcinoma [17]. Various histopathological diagnoses were reported: LAMN in five cases (45.45%), neuroendocrine tumor in three (27.27%), and adenocarcinoma, diffuse large B-cell lymphoma, and fibroma in one case each. Our presented patient exhibited a prolonged duration of inguinal hernia, complicated by soft tissue involvement of the abdominal wall, leading to the choice of an open approach. The decision to proceed with the right hemicolectomy was based on the colon damage in the context of emergency surgery for abdominal sepsis rather than for oncological considerations. Given the histopathological findings, no adjunctive oncological interventions were required.

Cases of AH in recurrent hernias are scarce. In a systematic review including 162 patients with AH [5], only 11 (6.8%) experienced recurrent inguinal hernias. None of the cases of AH coexisting with appendiceal tumors were associated with recurrent hernias. The case presented here had a previous history of laparoscopic inguinal mesh repair using polypropylene mesh and metallic tackers for fixation.

The pathophysiology underlying appendicitis within the hernia sac is thought to stem from incarceration, followed by periappendicular inflammation and adhesion formation of the appendix to the hernia sac. This, combined with contractions of the abdominal wall, leads to persistent inflammation, ischemia of the appendix, edema, venous stasis, and, eventually, appendiceal necrosis [17]. In the presented case, the appendix's incarceration may have been facilitated by two factors: 1) The presence of prosthetic material from a previous inguinal repair, which can generate significant adhesions within the hernia sac, especially with the use of metallic tackers, and 2) the likely desmoplastic reaction that some tumors can present, resulting to adhesions to adjacent tissues.

## Conclusions

Individually, AHs and appendiceal neoplasms are infrequent occurrences. Their simultaneous presentation, associated with perforation and within a region of prior inguinal repair, had not been previously documented or published in the consulted literature.

The chronic inflammation induced by prosthetic material, alongside neoplastic activity, may contribute to the adhesion of the appendix to the hernia sac, leading to an insidious progression resulting in complicated acute appendicitis.
